# MODIFIABLE AND NON-MODIFIABLE RISK FACTORS FOR TUBERCULOSIS AMONG ADULTS IN INDONESIA: A SYSTEMATIC REVIEW AND META-ANALYSIS

**DOI:** 10.21010/Ajidv18i2.3

**Published:** 2024-03-08

**Authors:** NINDREA Ricvan Dana, SUSANTI Rika, INDIKA Pudia M, MAISA Benny Alexander, SUKMA Muthia, ROSALINA Linda, WIDYA Astri, TAUFIQA Zuhrah, AGUSTIAN Dede Rahman, FITHRIA Rahmi, PUTRI Nomira, NINGSIH Dianni Arma Wahyu Setia, LUBIS Bella LucintaRillova Arif, MARDIAH Ainil, EZEDDIN Maudy Octarini, LINDA Nova, MARISA Yosa Tamia, RAHMI Afriyeni Sri, SARI Anggun Permata, OKTAVIANA Mimin, HUMANI Flori Puspa, AMSAL Mochammad Fariz

**Affiliations:** 1 Department of Medicine, Faculty of Medicine, Universitas Negeri Padang, Bukittinggi, 26181, Indonesia; 2 Department of Forensic and Medicolegal, Faculty of Medicine, Universitas Andalas, Padang, Indonesia, 25127

**Keywords:** Adults, Modifiable, Non-modifiable, Tuberculosis, Indonesia

## Abstract

**Background:**

Controlling tuberculosis (TB) determinant factors in Indonesia is one way to control TB in the community. A review is needed to explore risk factors for TB in Indonesia as the key strategies for accelerating the TB preventive program.

The purpose of this review was to determine modifiable and non-modifiable risk factors for TB among adults in Indonesia.

**Materials and Methods:**

A meta-analysis was undertaken to review current studies related to modifiable and non-modifiable risk factors for TB among adults in Indonesia. A search of PubMed, ProQuest, and Google Scholar for related articles published (January 2000 until December 2023). The Pooled Odds Ratio (POR) from the acquired data were calculated with a 95% CI. The fixed and random effects analysis was performed. The results were presented as forest plots, and Begg’s test and Egger’s test were used to examine study bias. Review Manager (RevMan) 5.4 and Stata 14.2 were used to process and analyze all of the data.

**Results:**

This study results revealed the POR of non-modifiable risk factor (family history of TB) for TB among adults in Indonesia was 6.08 (95% CI 2.99-12.34). Based on modifiable risk factors, it is known that household contact have the highest POR (6.01, 2.57-14.04), followed by malnutrition (5.86, 2.50-13.69), inappropriate ventilation (5.57, 1.74–17.86), diabetes mellitus (4.92, 3.04-7.96), smoking behavior (3.24, 2.22-4.72), and low-income level (2.34, 1.42-3.87).

**Conclusion:**

Based on significant factors that are related to TB incidence, the results of this review may be valuable to the government in identifying the optimal strategy for TB prevention among adults.

## Introduction

An important global health concern is tuberculosis (TB) (Adane *et al.*, 2020). More than 1 million annual deaths are recorded from TB. TB illness continues to be a serious public health issue that affects people of all ages (Jiang *et al.*, 2022). The action plans have already established a new aim to eliminate TB epidemics by 2030, in which endemic nations must make further efforts to stop or lessen the effect of TB (Jeremiah *et al.*, 2021).

In a World Health Organization (WHO) report from 2020, Indonesia had the second-highest percentage of worldwide TB incidence (8.5%) (Jiang *et al.*, 2022). A strategy was created by the Indonesian government to eradicate TB (Jeremiah *et al.*, 2021). Controlling TB determinant factors is one way, thus keeping track of these elements in society is important for informing policymakers as they create a preventative program (Sulistyawati *et al.*, 2021).

TB has multiple causes. There are currently a number of recognized TB risk factors (Sulistyawati *et al.*, 2021). Low level of economic status, smoking, alcohol use, diabetes mellitus, HIV infection, malnutrition, contact history, exposure to silicosis, and ventilation condition are classified as modifiable risk factors (Jubulis *et al.*, 2014). Age, gender, and family history are non-modifiable risk factors (Sadeghi *et al.*, 2022; Bath *et al.*, 2017).

Previous study found the modifiable risk factors contributed for TB were smoking and inappropriate ventilation condition (Sulistyawati *et al.*, 2021). One study stated nutritional status, family history of TB and smoking as modifiable risk factors associated for TB (Nur *et al.*, 2022). Other studies revealed malnutrition, diabetes mellitus, smoking, alcohol consumption, TB contact and poor families as modifiable risk factors for TB (Destiany *et al.*, 2020; Fibriana *et al.*, 2020; Stang *et al.*, 2020). Non-modifiable risk factors, namely family history of TB and age were associated for TB (Fibriana *et al.*, 2020; Nur *et al.*, 2022). However, conflicting findings regarding the significance of age and sex as risk factors for TB exist (Destiany *et al.*, 2020; Stang *et al.*, 2020), likely due to variations in study populations and methodologies.

The novelty of this study lies in its focus on synthesizing existing knowledge of both modifiable and non-modifiable risk factors for TB specifically among adults in Indonesia. While previous studies have examined individual risk factors, there is a lack of a comprehensive review that consolidates and analyzes these findings within the context of Indonesia. By undertaking this review, the study aims to provide a more nuanced understanding of how these risk factors interact within the unique socio-cultural and environmental landscape of Indonesia. This approach is crucial for developing targeted and effective TB preventive programs tailored to the country’s specific needs. Furthermore, the study seeks to address discrepancies in previous research findings by critically evaluating the methodologies and populations involved, thereby contributing to the advancement of knowledge in the field of TB epidemiology and public health intervention.

## Materials and Methods

### Study design and research sample

A meta-analysis was undertaken to review current studies related to modifiable and non-modifiable risk factors for TB among adults in Indonesia. This study follows the Preferred Reporting Items for Systematic Reviews and Meta-Analysis (PRISMA) guidelines (Page *et al.*, 2021).

### Eligibility criteria

Only original publications with a case-control or cohort study design, English language, and human participants as study subjects were included. Exclusion criteria for the study encompassed unavailability of a full-text version, inappropriate topics, and data from articles that could not be used for further examination.

### Search approach and study collection

A search of PubMed, ProQuest, and Google Scholar for related articles published (January 2000 until December 2023) with four main keywords “adult” AND “risk factors” AND “tuberculosis” AND “Indonesia”. In this study, tuberculosis was the outcome variable, while the exposure variables comprised modifiable and non-modifiable risk factors. Two independent investigators conducted the literature search. After the initial search, duplicates were manually eliminated, and the titles/abstracts were screened for relevance. The full texts of potential articles were then assessed using the criteria.

### Data extraction

**Figure 1:**
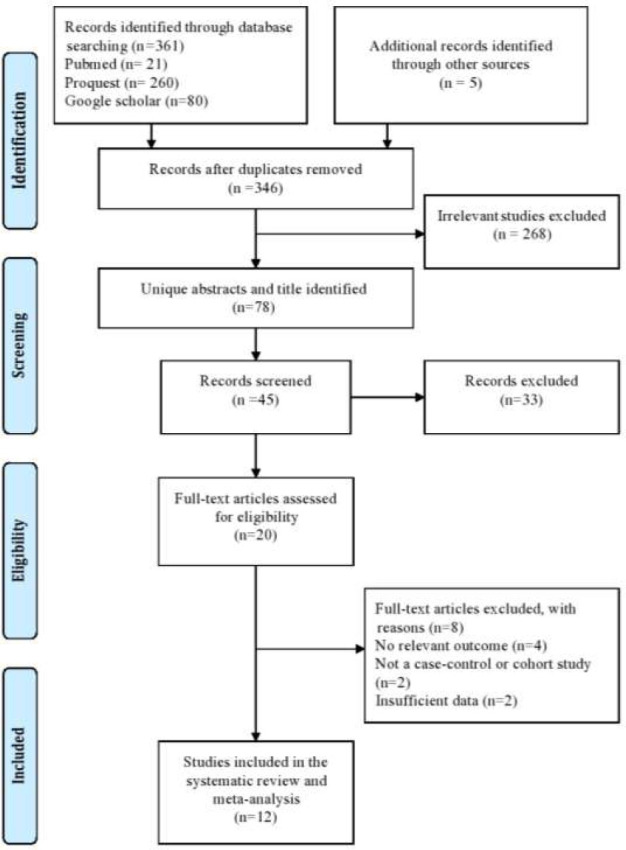
PRISMA flowchart.

Two different authors used structured extraction forms to obtain data. The processes of searching for research articles were depicted using PRISMA flowcharts (Figure 1).

**Figure 2:**
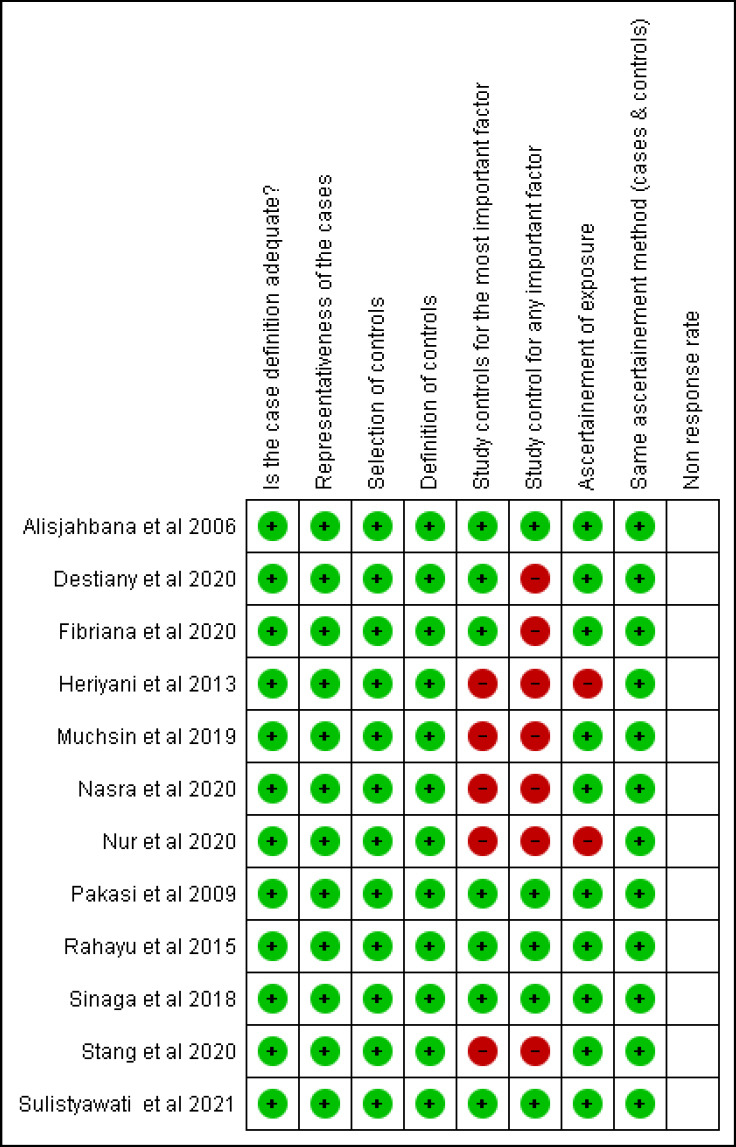
The quality of the publications using the Newcastle-Ottawa Quality Assessment Scale (NOS).

The quality of the publications was evaluated using the Newcastle-Ottawa Quality Assessment Scale (NOS). The NOS assessed nine questions: 1) Is the case definition sufficient? 2) Are the cases representative? 3) Selection of controls; 4) Definition of controls; 5) Study controls for the most significant factor; 6) Study controls for any significant factor; 7) Exposure measurement; 8) Identical measurement procedure (cases and controls); 9) Non-response rate. Articles were categorized into low, medium, and high-quality groups using the numbers 0–3, 4–6, and 7–9 (Figure 2) (Gusnedi *et al.*, 2023).

### Data analysis

The pooled stunting prevalence and the Pooled Odds Ratio (POR) from the acquired data were calculated with a 95% confidence interval (CI). I^2^ indicates that there was heterogeneity between publications if it was greater than 50%. If the outcome was heterogeneous, the random effect analysis was performed, and if it was homogeneous, the fixed-effect analysis was utilized. Furthermore, the findings were presented as forest plots, and Begg’s and Egger’s tests were used to identify study bias (Nindrea *et al.*, 2018). There was no publication bias among the studies, according to the p > 0.05 findings of the two tests. Review Manager (RevMan) 5.4 and Stata 14.2 were used to process and analyze all of the data.

## Results

Twelve current studies were considered in this systematic review research on modifiable and non-modifiable risk factors for TB among adults in Indonesia (Alisjahbana *et al.*, 2006; Pakasi *et al.*, 2009; Heriyani *et al.*, 2013; Rahayu *et al.*, 2015; Sinaga *et al.*, 2018; Muchsin *et al.*, 2019; Destiany *et al.*, 2020; Fibriana *et al.*, 2020; Nasra *et al.*, 2020; Stang *et al.*, 2020; Nur *et al.*, 2020; Sulistyawati *et al.*, 2021) (Table 1).

**Table 1:** Systematic review of modifiable and non-modifiable risk factors for tuberculosis among adults in Indonesia.

First author, year	Year of study	Region	Study design	Total samples	Characteristics	Risk factors
Alisjahbana *et al*	2006	Central Jakarta and Bandung	Case-control study	1,010	Age of cases and control (median 30 y); male, 52%; BMI of cases vs control (17.7 vs 21.5 kg/m^2^)	Diabetes mellitus and household contact
Pakasi *et al*	2009	Timor and Rote Islands	Case-control study	492	Mean age (30 y); Sex (M, 56.3%; F, 43.7%); BMI of cases vs control (16.1 vs 19.4 kg/m^2^)	Family history of TB and malnutrition
Heriyani *et al*	2013	Banjarmasin, Kalimantan	Case-control study	154	Sex (55.84% were male); age (35-<45 years were 25.97%)	Low income level, smoking behavior, inappropriate ventilation
Rahayu *et al*	2015	Semarang District	Case-control study	212	The mean age case and control was 41.2±15.3 and 35.7±11.7 years; Sex (M vs F were 50%)	Low income level
Sinaga *et al*	2018	Medan	Case-control study	200	Aged 16-55 years; male of cases vs control (70.0% vs 70.0%)	Smoking behavior
Muchsin *et al*	2019	Langsa, North Sumatera	Case-control study	232	Sex (M vs F were 50%)	Malnutrition, and inappropriate ventilation
Destiany *et al*	2020	Makassar	Case-control study	90	45-54 years (cases, 37.78%; control, 35.56%); male (cases, 62.22%; control, 68.89%); high school educational background (cases, 37.78%; control, 37.78%)	Low income level and malnutrition
Fibriana *et al*	2020	Semarang	Case-control study	75	15-54 years (cases, 86.7%; control, 88.9%); male (cases, 53.3%; control, 44.4%)	Diabetes mellitus and household contact
Nasra *et al*	2020	South Sulawesi	Case-control study	102	Productive age (cases, 79.4%; control, 79.4%); male (cases, 58.8%; control, 58.8%)	Family history, household contact, low income level, inappropriate ventilation
Stang *et al*	2020	Makassar	Case-control study	120	26-33 years (cases, 24.5%; control, 22.9%); low level of education (cases, 83.7%; control, 70.8%)	Household contact
Nur *et al*	2020	Jeneponto District, South Sulawesi Province	Case-control study	147	Productive age (cases, 68.3%; control, 71.7%); male (cases, 71.7%; control, 55.0%)	Malnutrition, smoking behavior and household contact
Sulistyawati et al	2021	Yogyakarta	Case-control study	69	Male (cases, 48.0%; control, 39.0%); 17-45 years (cases, 74.0%; control, 70.0%)	Family history of TB, smoking behavior, inappropriate ventilation
Total samples				2,903		

**Abbreviation:** BMI, body mass index; M, male; F, female; CI, confidence interval; NOS, Newcastle–Ottawa Quality Assessment Scale

The total sample from the included studies was 2,903 participants^4,8-11,14-20^. This study that revealed non-modifiable risk factors for TB among adults in Indonesia were a family history of TB, and modifiable risk factors (low income level, household contact, inappropriate ventilation, smoking behavior, diabetes mellitus, and malnutrition).

**Figure 3:**
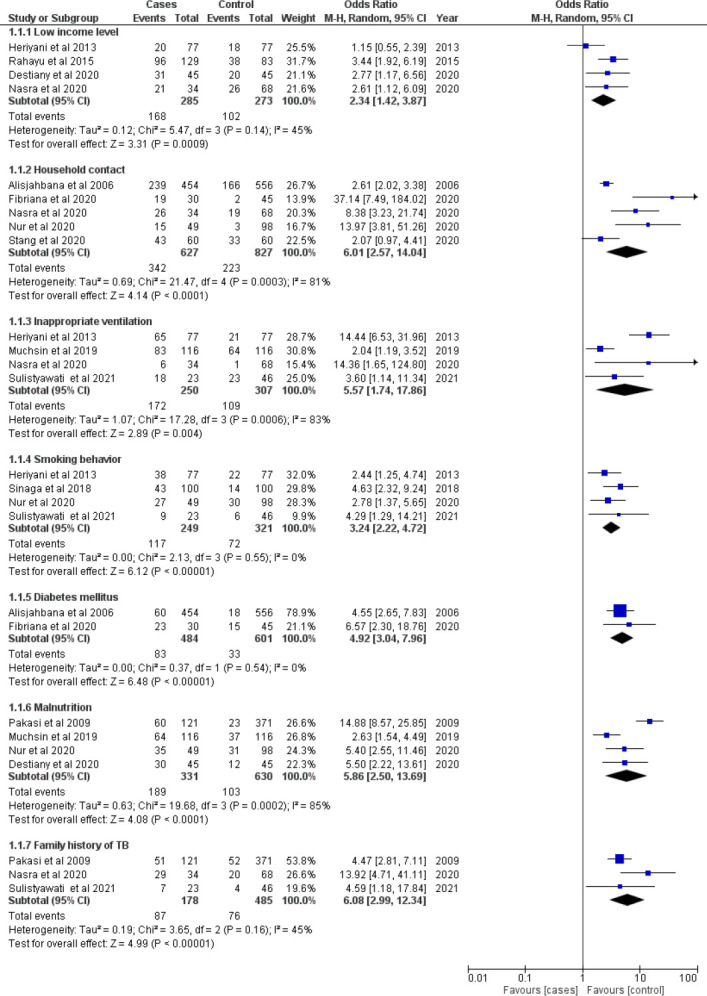
Meta-estimate of modifiable risk factors and non-modifiable risk factor (family history of TB) for TB among adults in Indonesia.

Meta-estimate of modifiable and non-modifiable risk factors for TB among adults in Indonesia (Figure 3).

Figure 3 revealed modifiable risk factors, it is known that household contacts have the highest POR (95% CI) (6.01, 2.57-14.04), followed by malnutrition (5.86, 2.50-13.69), inappropriate ventilation (5.57, 1.74–17.86), diabetes mellitus (4.92, 3.04-7.96), smoking behavior (3.24, 2.22-4.72), and low-income level (2.34, 1.42-3.87). The heterogeneity analysis revealed homogenous in low-income level, smoking behavior, and diabetes mellitus for TB among adults in Indonesia (I^2^ ≤ 50%). However, heterogeneity in household contact, inappropriate ventilation, and malnutrition for TB among adults in Indonesia (I^2^ > 50%).

The non-modifiable risk factor (family history of TB) for TB among adults in Indonesia was 6.08 (95% CI 2.99-12.34). The heterogeneity analysis revealed homogeneity of family history of TB for TB among adults in Indonesia (I^2^ ≤ 50%).

The results of Egger’s test to assess bias among studies are included (Table 2).

**Table 2:** Begg’s test and Egger’s test for small-study effects among modifiable and non-modifiable risk factors for tuberculosis among adults in Indonesia

Variables	Test for small-study effects	

	Begg’s test (p-value)	Egger’s test (p-value)
**Non-modifiable**		
Family history of TB	0.450	0.590
**Modifiable**		
Low income level	0.510	0.688
Household contact	0.055	0.085
Inappropriate ventilation	0.511	0.482
Smoking behaviour	0.602	0.807
Diabetes mellitus	0.411	0.500
Malnutrition	0.878	0.988

It revealed that non-modifiable factors (family history of TB), and modifiable factors (low-income level, household contact, inappropriate ventilation, smoking behavior, diabetes mellitus, and malnutrition) showed no study bias among the included publications (p>0.05).

## Discussion

In this study, we found that modifiable risk factors for TB among adults in Indonesia, it was also revealed that household contacts have the highest POR (6.01). Household contacts were three times more likely to develop TB than individuals who lived in adequate-sized rooms. This is consistent with earlier studies conducted in Ethiopia, Pakistan, and sub-Saharan Africa (Hargreaves et al., 2011; Kirenga *et al.*, 2015; Khaliq *et al.*, 2015). This could be connected to cramped conditions and inadequate airflow, which can hasten the spread of TB (WHO, 2009). However, in a society with extended families, the contact network goes beyond the nuclear family despite the fact that the index case had many home contacts (WHO, 2009; Hargreaves et al., 2011). The balance of TB transmission, including whether it happens largely in homes or in the population as a whole, is unknown and may vary from region to region based on the frequency of the disease and the mixing patterns of infectious cases (Hargreaves et al., 2011). The location of TB transmission can have a significant impact on TB control strategies. In Indonesia, the limitations of the present diagnostic techniques for early or minimal disease make it difficult to evaluate interactions within households. Improved diagnostic techniques and assessments of the dangers associated with the household environment will be needed for new household contact tracing strategies.

According to our study, malnutrition increased the risk of TB by 5.86 times. Previous study stated dietary conditions and the prevalence of TB are related. Malnutrition will reduce the immune system’s ability to fight disease, making a person with poor nutritional status more prone to contracting TB. Another study found micronutrient deficits and protein-energy deficiency raise the risk of TB. Malnourished TB patients have been shown to have slower recovery times and greater fatality rates than well-nourished individuals (Gupta *et al.*, 2009). In Ethiopia, the prevalence of undernutrition among adult TB patients was severe (Muse *et al.*, 2021). The likelihood of malnutrition was 3.23 times higher in TB patients over the age of 25 years. This conclusion is consistent with one from Ghana (Dodor *et al.*, 2008). This is as a result of the fact that co-morbid illnesses, such as chronic diseases, carry increasing risks as people age (Nunes *et al.*, 2016). In Indonesia, a large percentage of patients with TB are malnourished. Especially among the indigenous populations who live in extended families, this undoubtedly plays a role in the development of TB. The local government is advised to create a Body Mass Index (BMI) based malnutrition screening program.

This study found that inappropriate ventilation also increased the risk of TB by 5.57 times. A common risk factor for TB is crowded living conditions, like shared housing (Lönnroth *et al.*, 2009; Lienhardt *et al.*, 2012). An earlier study found that TB exposure, incidence, and treatment adherence were all correlated with the affordability and quality features of substandard housing. Additionally, we discovered that practically all eight stages of TB development and its effects were connected to inadequate housing. The prevalence of homelessness, which was closely associated with TB infection, is rising globally (Lönnroth *et al.*, 2009).

In this study diabetes mellitus was associated with TB among adults with POR = 4.92. Diabetes mellitus (DM) has been linked to TB in previous studies (Lönnroth *et al.*, 2009; Fibriana *et al.*, 2020). Another study found that 14.8% of the incidence of TB in India was caused by DM. According to a review of recently published reports, the odds ratio for DM as a risk factor for tuberculosis varied in different regions, ranging from 1.23 to 6 (Gupta *et al.*, 2009). The high correlation found in our study between DM and TB may have a possible explanation. Indonesia has a young population and is still in development (Harahap *et al.*, 2017). DM as a comorbidity with TB must therefore be disregarded. The discovery that DM is a significant risk factor for TB in our location has clear implications for TB control. High attention must be given to DM control programs in the population, especially young people and TB patients, due to the high prevalence of DM in TB patients.

We also found smoking behavior increased the risk of TB by 3.24 times. A higher risk of tuberculosis was also demonstrated by prior studies of cigarette use and chronic respiratory disease (Lönnroth *et al.*, 2009; Nunes *et al.*, 2016). There was no statistically significant difference in TB prevalence between cigarette smokers and those with chronic bronchitis. According to another study, cigarette use is linked to TB, and it is more common in men than in women. Smokers are more likely to develop TB than non-smokers (OR = 1.8). Additionally, the current study found a favorable relationship between tobacco usage and smoking for TB among adults (Khaliq *et al.*, 2015). One of the highest smoking rates in the world is found in Indonesia (Jiang *et al.*, 2022). Therefore, the task is not just to reduce the number of smokers but also to reduce the number of TB cases among smokers. To help a person stay smoke-free, smoking cessation messages must both prevent TB and continue after TB treatment. Healthcare professionals, providers, and family members are in an excellent position to support the doctor’s advice on quitting smoking and assist individuals who are smokers in doing so.

This study also revealed low income level associated with TB among adults with POR = 2.34. According to a study from India (Bhat *et al.*, 2017), poor people had a high frequency of incomplete TB knowledge. According to a study from Ethiopia (Dodor *et al.*, 2008), those with low incomes tend to be less health-conscious. Economic status is related to access to health facilities, the ability to meet nutritional needs, and access to a good and standardized home environment (Khaliq *et al.*, 2015). These factors all increase the likelihood of TB. Therefore, the government must take into consideration measures to raise income by encouraging the informal sector to engage in entrepreneurship and giving capital support to enhance their welfare. Such a gain in income will be consistent with an increase in the ability to purchase nutritious food, the availability of housing, and access to high-quality healthcare services.

This study found that family history of TB is a non-modifiable risk factor for TB among adults in Indonesia (POR = 6.08). According to a prior study in Uganda, 17.5% of TB patients reported having TB in their families. Family history is a well-known TB risk factor (Kirenga *et al.*, 2015). Household contacts with a family history of TB were more likely to get the disease than those without such a history. This was similar to studies conducted in India and Ethiopia (Bhat *et al.*, 2017; Nasra *et al.*, 2020). People who have a prior history of TB in their families should have early screening and health examinations at medical facilities, such as hospitals or primary health care centers, on the risks they may have. In order to carry out successful preventative initiatives. In addition, there is a need for awareness, knowledge, and comprehension of other TB risk factors.

The Indonesian government developed a plan to end TB. Monitoring such factors in society is crucial for informing policymakers as they develop a preventative program since controlling TB determining factors is one method. Indonesia’s TB control policy is implemented through a health sector and multi-sector approach using a variety of risk factor management strategies, with a focus on improving individual health levels and reducing TB infection in public areas. To help Indonesia reach its 2030 TB elimination target, the central and local governments’ roles in TB control must be increased. One of them is the health reform, which highlights the value of sustainable implementation in Indonesia at the regional level (Nindrea *et al.*, 2020; Jiang *et al.*, 2022; Nindrea *et al.*, 2023).

To the best of our knowledge, this study is the first meta-analysis of both modifiable and non-modifiable risk factors for TB among adults in Indonesia, providing a comprehensive overview of the current knowledge in this area. We revealed the POR for modifiable and non-modifiable risk factors with TB among adults identified from a diverse range of studies with large sample size and different demographic characteristics in the country. Thus, the analysis could provide a basis for concluding risk factors statistically associated with the incidence of TB among adults in Indonesia. However, risk factors were estimated by odds ratio and it may be affected by other confounding variables. The other limitation is that the search strategy restricted articles published in the selected databases and only in the English language. There might be articles published in other databases using another language that was not included. Furthermore, the study focuses specifically on adults in Indonesia, and the findings may not be generalizable to other age groups or populations in different geographic regions.

The implications of this study may be valuable to the government in identifying the optimal strategy for TB prevention among adults. Additionally, health promotion and education regarding the prevention of TB based on modifiable and non-modifiable risk factors must be carried out. Health professionals and capable health volunteers can provide community- or individual-based promotion and education from home to home. For the public to understand TB preventive initiatives, it is crucial to disseminate this information via the right promotional media, both online and offline.

## Conclusion

This review revealed family history of TB as a non-modifiable risk factor for TB among adults in Indonesia and household contact, malnutrition, inappropriate ventilation, diabetes mellitus, smoking behavior, and low income level are all modifiable risk factors for TB. The findings of this study may be helpful to the government in determining the best plan for TB prevention among adults based on significant factors that are related to TB incidence. The prevention and control of TB based on modifiable and non-modifiable risk factors requires health promotion and education. From home to home, qualified health volunteers and health professionals can offer community- or individual-based promotion and education, for the general public to comprehend TB prevention efforts.
